# Clavicular Chondrosarcoma: A Case Report and Brief Review of the Literature

**Published:** 2016-07-01

**Authors:** Ali Ghorbani Abdehgah, Behnam Molavi, Saeed Reza Mehrpour, Amir Reza Radmard, Mohammad Mahjori, Nasser Kamalian, Hosein Kamranzadeh

**Affiliations:** 1 Thoracic Surgeon, Assistant Professor, Department of Surgery, Research Center for Improvement of Surgical Outcomes and Procedures, Shariati Hospital, Tehran University of Medical Sciences, Tehran, Iran; 2 Vascular Surgeon, Assistant Professor, Department of Surgery, Research Center for Improvement of Surgical Outcomes and Procedures, Shariati Hospital, Tehran University of Medical Sciences, Tehran, Iran; 3 Orthopedic Surgeon, Assistant Professor, Department of Orthopedics, Shariati Hospital, Tehran University of Medical Sciences, Tehran, Iran; 4 Radiologist, Assistant Professor, Department of Radiology, Shariati Hospital, Tehran University of Medical Sciences, Tehran, Iran; 5 General Surgeon, Department of Surgery, Research Center for Improvement of Surgical Outcomes and Procedures, Shariati Hospital, Tehran University of Medical Sciences, Tehran, Iran; 6 Pathologist, Professor, Department of Pathology, Shariati Hospital, Tehran University of Medical Sciences, Tehran, Iran; 7 Medical Oncologist, Assistant Professor, Hematology-Oncology and Stem Cell Transplantation Research Center, Tehran University of Medical Sciences, Tehran, Iran

**Keywords:** Clavicular, Chondrosarcoma, Primary

## Abstract

Clavicular bone tumors occur in less than 0.5 percent of bone tumors. Primary chondrosarcoma is very rare even among clavicle tumors. The main symptom is a touchable mass in 69 % of patients. Dedicated centers using FNA and cytology can reach a correct diagnosis in 94% of cases. Treatment planning is done using simple X-ray, CT-scan, shoulder MRI, chest CT-scan and whole body technetium scan. Treatment of choice for primary chondrosarcoma of clavicle is surgical resection.

## Introduction

 Tumors of clavicle are rare; most of them are primary malignant lesions.^   1 ^^-^   3  Chondrosarcomas mostly arise from pelvis and trunk bones; primary chest wall chondrosarcomas are relatively rare.^   4 ^^,^^   5 ^

Chondrosarcomas are more prevalent in adults than in children (being more common in males than females), they are more common in people older than 40. They occur mainly in pelvis, femur, homerus and scapula.^   6 ^ Chest wall chondrosarcomas originate from ribs in 80% of cases, the rest arises from sternum. Clavicle and scapula are less likely to be the origin of chondrosarcoma.^   7 ^ Since Chondrosarchomas do not respond well to radiotherapy or chemotherapy, surgery is preferred. In clavicle malignancies, where the goal is definitive treatment, total claviculectomy is the best choice. 2^,^^5^^,^^7^ 

## CASE REPORT

 The patient was a 22 year old woman who was referred to the surgery ward early in the February 2014 from the orthopedic ward for multidisciplinary surgery. Patient had a mass in anterior thorax and clavicle, discovered 6 months earlier. It had grown steadily and was not painful at first but the patient complained of pain at the time of referral. She had no history of alcohol or drugs, nor did she have a history of smoking. She had no family history of known diseases.

In examination, a large mass with the dimensions of 10*6 centimeters was discovered, taking up two thirds of the clavicle. Head, neck, heart, lungs, organs and the pulse were normal in physical examination.

**Needle biopsy**

The patient underwent core needle biopsy and was diagnosed as grade II chondrosarcoma.

**CT scan and MRI**

**Figure 1:**
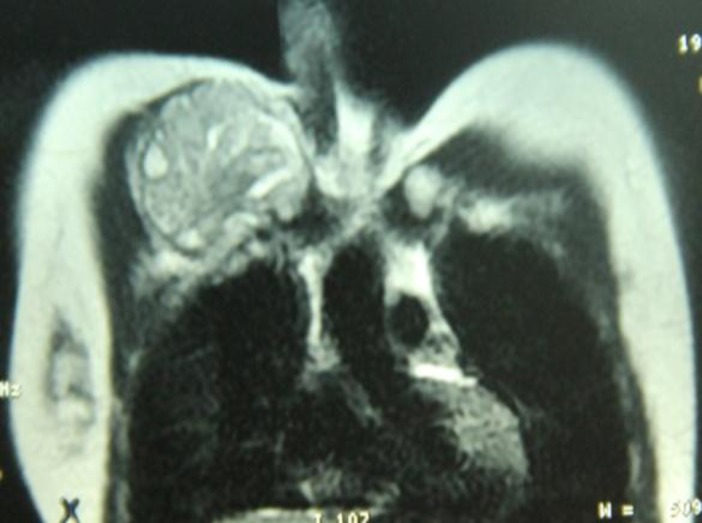
Coronal T2-weighted MRI shows a lobulated well-defined mass in right anterior chest wall arising from medial clavicle with bony destruction and upward extension to supraclavicular region consistent with chondrosarcoma.

In CT scan and MRI, a 91*57*59 millimeters mass was observed, proximal. There was no metastasis (Figure 1).

Due to the tumor’s situation and its proximity to neurovascular network, the patient was sent to Shariati Hospital's surgery ward to be operated on by a multidisciplinary team consisting of thoracic, vascular, orthopedic surgeons. Decision on surgical resection of the mass was made after a review meeting between the mentioned surgical team and oncologists.

**Pathology report**

**Figure 2:**
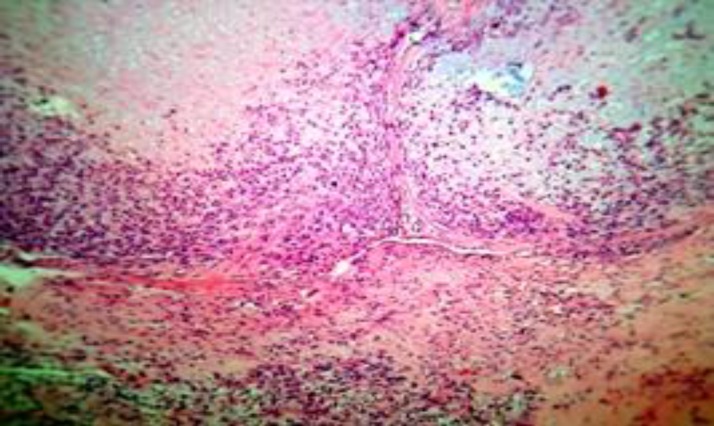
Microscopic view of the lesion showing lobulation in the central part of the hyaline cartilage at the periphery of en differentiated mesenchymal cells can be seen

**Figure 3:**
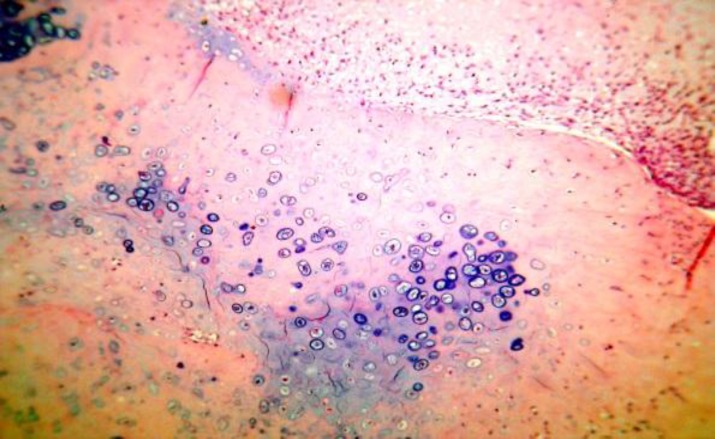
A microscopic area of hyaline cartilage with calcification, non-uniform distribution of Lacuna and cartilage atypical cells

Pathologic stage is definitely important in prognosis, grade I chondrosarcoma has low malignant nature while grade II and grade III chondrosarcoma are high malignant. Five year survival in grade I is 90%, it falls to 60% for grades II and III.^   6 ^ In the aforementioned patient the chondrosarcoma was grade II (Figure 2 and 3).

## Discussion

 Chondrosarcoma is a malignant tumor result of neoplastic chondrogenesis.^   5 ^ It often arises from pelvis and long bones. Chest wall chondrosarcomas may not cause pain, which results in delayed diagnosis. One reason for painlessness is that the bones in the thorax are not weight bearing bones.^   8 ^ In the mentioned patient there was no pain at first, and only slight pain reported at the time of examination.

The other reason for delayed diagnosis is FNAC (needle biopsy) not giving definite answers; in non-specialized centers only 26% of diagnosis is accurate. In specialized centers this number rises to 94%.^   8 ^ In our case, final pathology confirmed the result of FNA. If the answer to needle biopsy is benign or indefinite, it should not be trusted, in such cases it is best to repeat the needle biopsy or revert to open biopsy.^   8 ^

In most cases, chondrosarcoma shows up as a radiolucent lesion with indistinctive edges, calcification is observed as well.^   9 ^ On CT, the density of chondrosarcoma is comparable to that of muscle with internal chondrosarcoma is comparable to that of muscle and calcification. MRI reveals cartilage matrix as low and high signal intensity on T1 and T2 weighted images, respectively. ^4^^,^^5^  In patients who undergo wide resection, less recurrence has been observed, recurrence mostly occurs in the first five years after surgery.^   10 ^

**Figure 4:**
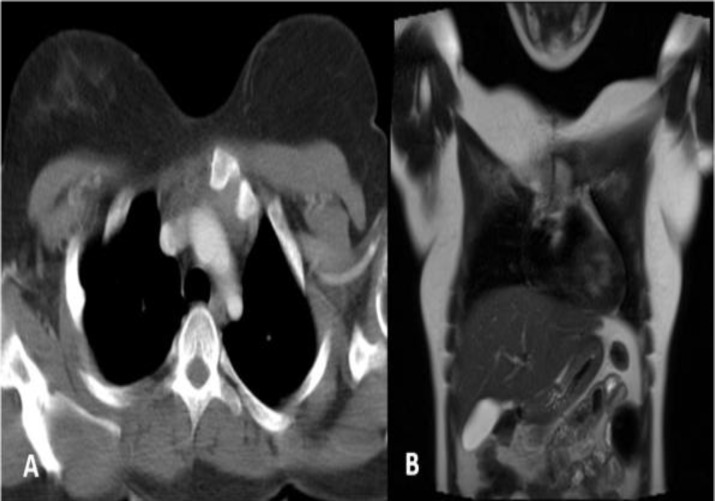
Post operative CT and MR images of the patients. There is no evidence of tumoral remnant in bed of right clavicular resection in axial CT (A) and coronal T2-weighted (B) images.

**Figure 5:**
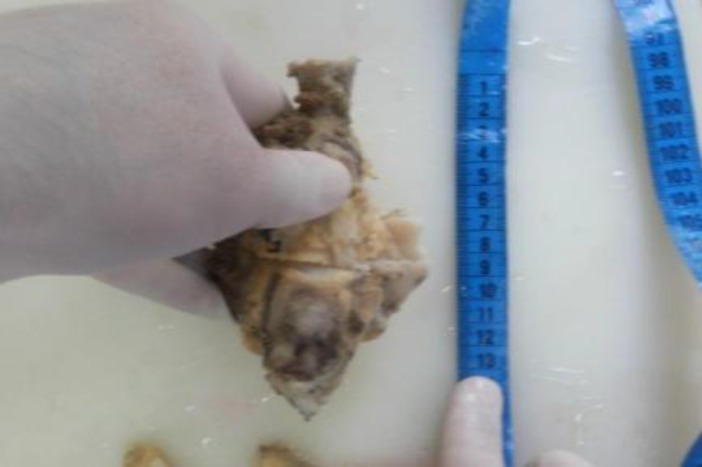
Clavicular mass after resection

**Figure 6:**
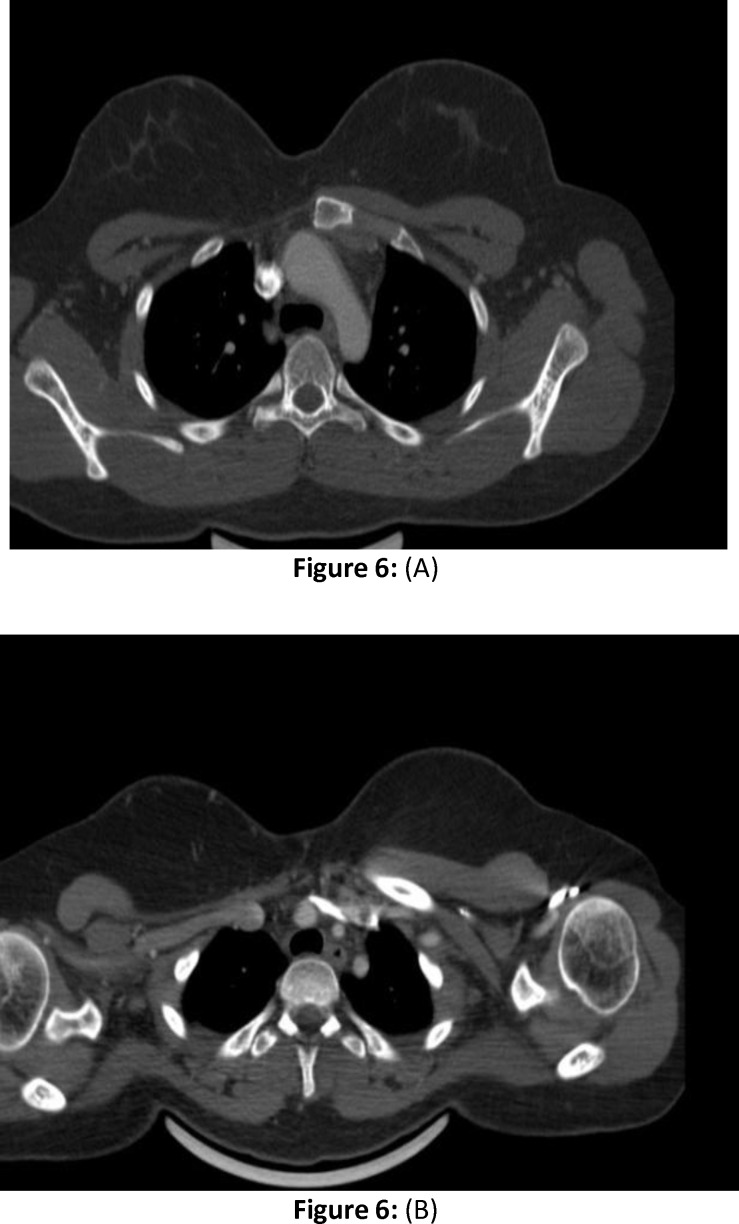
Post operative axial CT scan (A and B) following treatment, which show no mass lesion in operated bed of right clavicular resection. Sternal margin is also smooth

Total claviculectomy was performed on our patient, a quarter of sternum was resected alongside the tumor due to the tumor's position (proximal position) and so that proximal margin and distal margin were free of tumor (Figure 4, 5 and 6).

Unlike Osteosarcoma and Ewing sarcoma, chondrosarcoma does not answer to chemotherapy and radiotherapy.^   11 ^ Total claviclectomy is the best treatment choice when the complete cure is intended.^   2 ^ Chondrosarcoma mostly occurs to older patients in areas such as pelvis, arm and thigh. The discussed patient was 22 year old and the tumor was in her clavicle.

**Follow-up**

One year after surgery and because of close margin, the patient was referred to medical and radiation oncologist for adjuvant chemotherapy and radiotherapy.

## CONCLUSION

 To summarize, in a patient with a clavicle tumor, needle biopsy's results should not be trusted even in the absence of pain, if the tumor comes up as benign, the test should be repeated. The main treatment for primary chondrosarcoma of clavicle is surgery.
